# Assessing the hidden risks of non‐thermal processing: Ultrasound‐induced toxicity of cashew apple juice in a zebrafish model

**DOI:** 10.1002/jsfa.70738

**Published:** 2026-05-27

**Authors:** Thaiz Batista Azevedo Rangel Miguel, Sergimar Kennedy de Paiva Pinheiro, Jorge Henrique de Andrade Vieira, Fabiano André Narciso Fernandes, Sueli Rodrigues, Emilio de Castro Miguel

**Affiliations:** ^1^ Biotechnology Laboratory, Food Engineering Department Federal University of Ceará Fortaleza Brazil; ^2^ Biomaterials Laboratory, Department of Metallurgical Engineering and Materials and Analytical Center Federal University of Ceará Fortaleza Brazil; ^3^ Chemical Engineering Department Federal University of Ceará Fortaleza Brazil

**Keywords:** food toxicity, developmental toxicity, food safety, *Danio rerio*, *Artemia salina*

## Abstract

**Background:**

High‐intensity ultrasound is widely advocated for the preservation of liquid foods; however, the potential formation or release of toxic compounds during processing remains under‐investigated. This study evaluated the toxicological profile of cashew apple juice processed by high‐intensity ultrasound (1250, 1875, and 2500 W;L⁻¹) using two biological models of differing complexity: brine shrimp (*Artemia salina*) and zebrafish (*Danio rerio*).

**Results:**

Neither unprocessed nor sonicated juice exhibited acute toxicity in *A. salina* nauplii. In contrast, *D. rerio* assays revealed significant, parameter‐dependent developmental toxicity in sonicated samples, whereas unprocessed juice proved biocompatible. Sonication induced severe morphological anomalies in zebrafish, including cardiac edema, spinal curvature, and growth retardation. Toxicity was inversely related to treatment duration; short processing times (5 min) induced higher mortality and morbidity than longer durations (15 min), suggesting the degradation of toxic compounds during extended processing.

**Conclusion:**

These findings demonstrate that *D. rerio* is a more sensitive model than *A. salina* for assessing the safety of non‐thermally processed foods in the tested conditions. Although ultrasound is effective, processing parameters can critically alter the toxicological profile of the food matrix, highlighting the need for comprehensive safety evaluations using sensitive vertebrate models prior to industrial application. © 2026 The Author(s). *Journal of the Science of Food and Agriculture* published by John Wiley & Sons Ltd on behalf of Society of Chemical Industry.

## INTRODUCTION

Non‐thermal food processing technologies, such as high‐intensity ultrasound, have attracted interest in the food industry for their ability to preserve the nutritional and sensory qualities of products such as fruit juices. By operating at lower temperatures, these methods usually prevent the degradation of vitamins and bioactive compounds that are often associated with traditional thermal pasteurization. However, the assumption that these ‘greener’ and ‘gentler’ technologies are inherently safer remains under‐scrutinized. The intense energy from processes such as sonication can induce chemical changes in the food matrix, potentially generating free radicals or altering the bioavailability of naturally occurring compounds.

Cashew apple (*Anacardium occidentale* L.) juice is a nutritionally rich beverage, valued for its high vitamin and phenolic compound content.^1^ However, this pseudo‐fruit, unlike the nut, has a short post‐harvest life.^2^ To overcome this limitation, the cashew pseudo‐fruit is processed into juice, extending its shelf life. Industrial thermal pasteurization during this process can lead to a reduction in its nutritional value.^1^, ^3^

The primary goal of contemporary food technology is to optimize the preservation of nutrients and food safety throughout the processing and storage stages.^4^ Cashew apple juice is considered safe and beneficial due to its high vitamin C content, antioxidants, and other nutrients. However, it also naturally contains potentially toxic substances, such as anacardic acids, which have demonstrated toxicity in animal studies at high doses.^5^ Cashew apple juice is also rich in tannins, which are polyphenolic compounds responsible for its astringent taste. Tannins have some health benefits but they are often considered to have antinutritional properties because they can bind with essential nutrients, such as proteins and minerals, making them less available for absorption and utilization in the body.^6^ Specific proteins in cashew apple, such as vicilin and legumin, can also cause allergic reactions in individuals sensitive to cashews. The cashew apple also contains urushiols, compounds that are structurally similar to the sensitizing compounds in poison ivy and poison oak.^7^ Ingestion of urushiols can cause systemic reactions.

Non‐thermal processing technologies, such as sonication, can be employed to prevent nutrient loss in cashew apple juice as an alternative to thermal processing.^4^, ^8^ Despite the wide application and benefits of ultrasound processing, this technology can generate free radicals in foods, which may have potentially toxic effects.^9^ Sonication can both disrupt plant cell structures and degrade chemical compounds; thus, the effects of sonication on toxicity may be non‐linear and dependent on power density and on processing time. The impact of ultrasound‐based food processing has already been shown to depend on the method and the chemical nature of the food matrix.^10^, ^11^, ^12^, ^13^

The effects of ultrasound on the physical and chemical properties of foods have been widely studied; however, there is a significant scarcity of research addressing the toxicological outcomes of these processed foods in biological systems. This knowledge gap is critical, as processing could inadvertently increase the concentration of natural toxins, posing a hidden food safety risk.

To address this gap, this study employed a dual‐model toxicological assessment. The brine shrimp (*Artemia salina*) was selected as a robust, widely used invertebrate model for screening acute lethality.^14^ Its widespread use in ecotoxicological studies is attributed to its adaptability to diverse test conditions, ease of laboratory cultivation, low cost, short life cycle, and high reproductive capacity.^15^ To provide a more accurate evaluation of developmental toxicity, the zebrafish (*Danio rerio*) embryo was selected. As a complex vertebrate model, the zebrafish offers several advantages, including transparent embryos that enable detailed morphological analysis and a genome that exhibits 70% similarity to human protein‐coding genes, making it a highly sensitive and relevant system for predicting potential human health risks.^16^, ^17^

The present work therefore aimed to evaluate the toxicity of ultrasound‐processed cashew apple juice on *A. salina* nauplii and zebrafish embryos. By comparing the responses of these two models, this study aimed to understand how sonication parameters affect the safety of cashew apple juice and to determine the level of biological complexity necessary for a comprehensive toxicological assessment of non‐thermally processed foods.

## MATERIALS AND METHODS

### Cashew apple juice

Non‐pasteurized frozen cashew apple pulp was acquired in Fortaleza, Brazil, (supplied by Nossa Fruta) and preserved at −20 °C. The fruit pulp was combined with water (1:3 w/v) in a food processor, and the juice was subjected to ultrasound treatment.

### Ultrasound processing

Samples (200 mL) were sonicated using a 19 kHz probe ultrasonic processor (model DES500; Unique, Indaiatuba, Brazil) equipped with a 13 mm titanium probe immersed 15 mm below the liquid surface. The treatments were performed at power settings of 250, 375, and 500 W (equivalent to ultrasonic power densities of 1250, 1875, and 2500 W L⁻¹) for durations of 5, 10, and 15 min. Following treatment, the samples were freeze‐dried and then stored at 4 °C until required for biological assays.

### Toxicity tests

#### Toxicity test applying *Artemia salina nauplii*

High‐quality *A. salina* cysts were provided by INVE Aquaculture Brazil (Fortaleza, CE, Brazil). The cysts were kept protected from light at low temperatures until they were used. To break dormancy, 0.2 g of cysts were exposed to 200 mL of artificial seawater (35 g L⁻¹ of sea salt) and incubated for 48 h at 24 ± 2 °C under a 16 h light and 8 h dark cycle.^18^ After 48 h, the nauplii were transferred to 24‐well plates containing artificial seawater for subsequent use in the experiments. Two concentrations (10 and 100 μg mL^−1^) of ultrasound‐processed cashew apple juice and unprocessed cashew apple juice were tested for 24 and 48 h. Two control groups were used: one exposed only to artificial seawater, and the other exposed to 0.5 mol L⁻¹ potassium dichromate (K_2_Cr_2_O_7_). The experiment was carried out in 24‐well plates with a final volume of 2 mL per well. Each group contained 50 individuals (five wells with ten individuals each). The ambient temperature was 24 ± 2 °C, and the same photoperiod used for cyst eclosion was applied. After 24 and 48 h, the dead individuals in each well were counted in a stereoscopic microscope (Zeiss Stemi 508 ‐ Zeiss, Jena, Germany). The experiment was considered valid when more than 90% of the individuals in the control group remained alive.^19^

#### Toxicity test applying zebrafish embryos

The zebrafish early life stages assay was performed according to the OECD guidelines on the fish embryo test (FET).^20^ Zebrafish eggs used in the study were provided by the Biomaterials Laboratory, Department of Metallurgical Engineering and Materials, Universidade Federal do Ceará, Brazil. The test began by exposing newly fertilized zebrafish eggs to 96 h postfertilization (hpf) at 28 ± 2 °C under a 16:8 (light:dark) photoperiod, with the selected concentrations of unprocessed and ultrasound‐processed cashew apple juice, including the control group. Two concentrations (10 and 100 μg mL^−1^) of ultrasound‐processed cashew apple juice and unprocessed cashew apple juice were tested. The extract dilution was performed in reconstituted water (medium). Twenty eggs per treatment were selected and distributed in 24‐well microplates. The experiment was performed in triplicate. Embryos and larvae were observed daily under a stereomicroscope (Stemi 508, Zeiss). The following parameters were evaluated: hatching, edema, malformations, mortality, body length, and yolk sac area. Malformations were scored when zebrafish presented anatomic abnormalities according to Kimmel guidelines.^21^ The ethics committee of the Universidade Federal do Ceará approved the zebrafish experiment (protocol CEUA no. 4 645 080 322).

### Statistical analysis

Data for the statistical analyses were collected at 24 and 48 h for *A. salina* nauplii and 24 and 96 h for zebrafish embryos. The results were expressed as multiple mean comparisons for each sample across different concentrations. A one‐way analysis of variance (ANOVA) and the Student's *t*‐test were used to determine the differences between treatments. Statistical significance was set at *P* < 0.05.

## RESULTS

### Toxicity in *Artemia salina* nauplii

**Figure 1:**
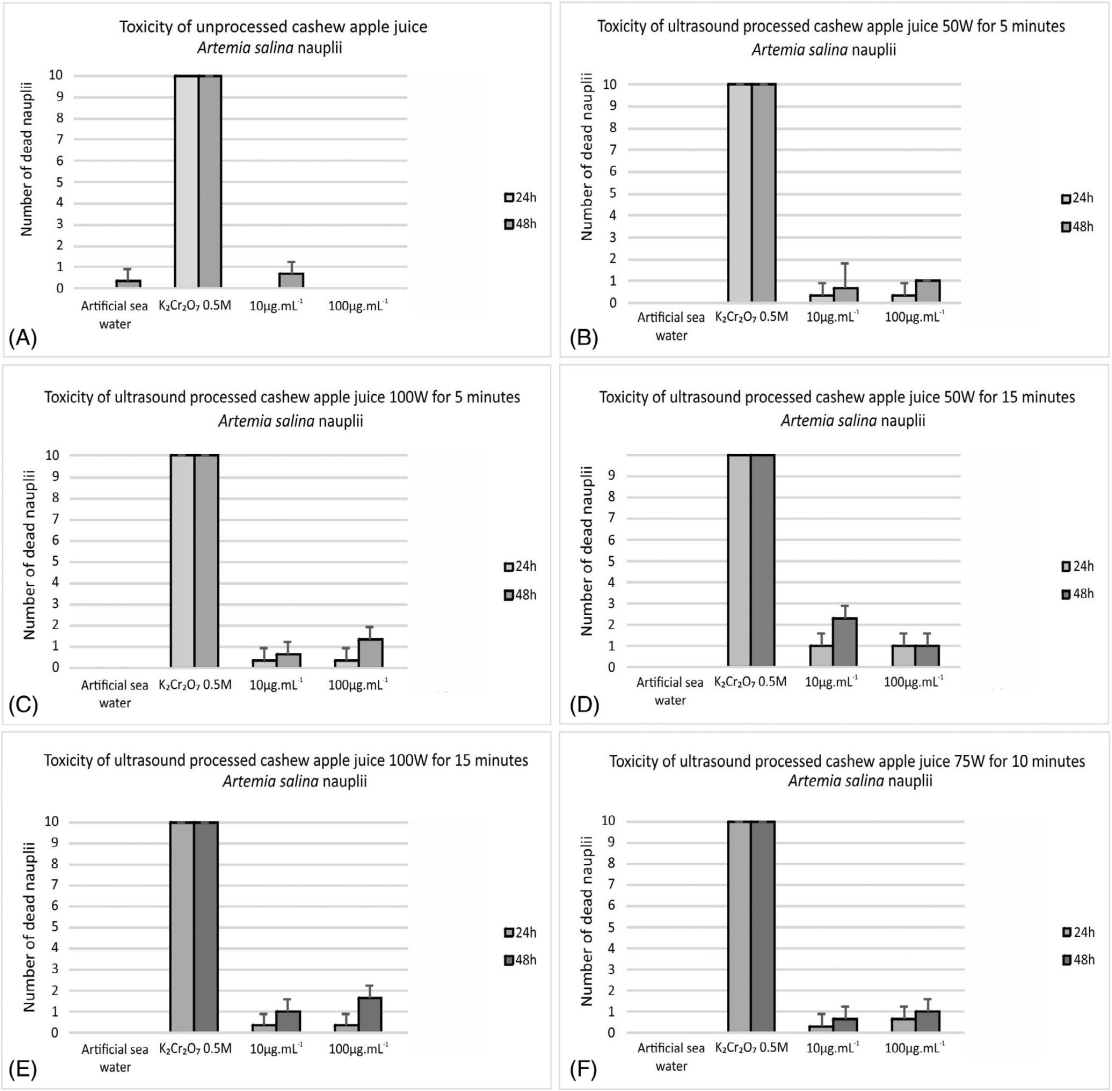
Acute toxicity of unprocessed cashew apple juice and ultrasound‐processed cashew apple juice (10 and 100 μg mL^−1^) in *Artemia salina* instar II nauplii at 24 and 48 h of exposure.

The cashew apple juice, both in its unprocessed form and after all ultrasound treatments, demonstrated no significant toxicity towards *A. salina* nauplii (Fig. 1). Across all tested power densities (1250, 1875, and 2500 W L⁻¹) and durations, mortality rates remained low and were not significantly different from the control group, indicating a lack of acute toxic effects from processing.

### Toxicity in zebrafish embryo

**Figure 2:**
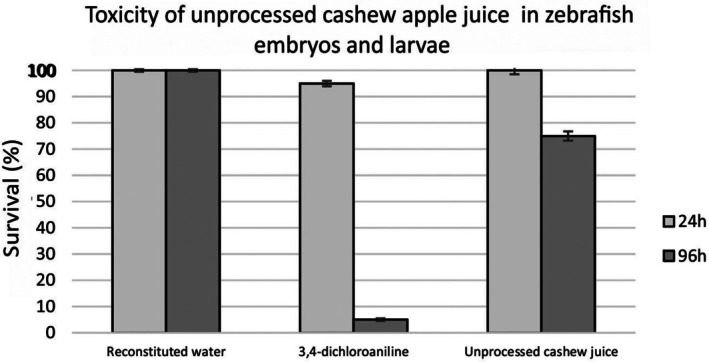
Acute toxicity of the negative control, positive control, and unprocessed cashew apple juice in zebrafish (*Danio rerio*) embryos and larvae at 24 and 96 h of exposure.

Before testing with cashew apple juice, the methodology was validated through the performance of the controls. The assay controls performed as expected, with the negative control (reconstituted water) showing 100% larval survival. In contrast, the positive control (3,4‐dichloroaniline) induced high mortality and developmental defects, resulting in only 5% larval survival after 96 h (Fig. 2), validating the test system.

**Figure 3:**
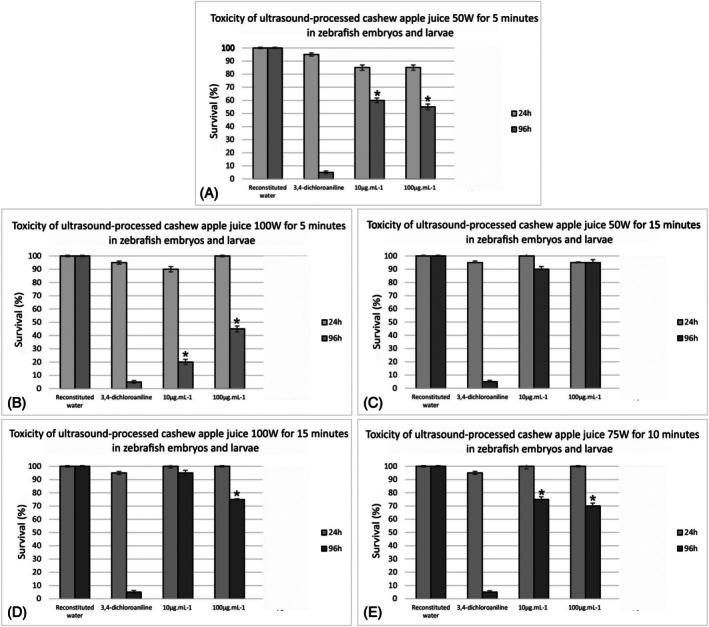
Acute toxicity of ultrasound‐processed cashew apple juice (10 and 100 μg mL^−1^) in zebrafish (*Danio rerio*) embryos and larvae at 24 and 96 h of exposure.

The unprocessed cashew juice exhibited a survival rate of nearly 100% within the first 24 h, identical to that of the negative control (reconstituted water). At 96 h, the survival rate for the unprocessed juice group decreased to approximately 75%. These results indicate that unprocessed cashew apple juice was largely biocompatible, showing no toxicity at 24 h and only a mild reduction in survival after 96 h of exposure (Fig. 3).

The toxicity of the ultrasound‐processed juice was time dependent (Fig. 3). No significant mortality was observed in zebrafish embryos at 24 h of exposure for either the 10 or 100 μg mL^−1^ concentrations, regardless of the processing condition. However, by 96 h, significant toxic effects emerged, with severity depending on the specific combination of sonication power density and duration. Short treatments (5 min) were notably toxic, with the 2500 W L⁻¹ condition reducing larval survival to as low as 25% at a concentration of 10 μg mL^−1^.

Longer processing times did not uniformly increase toxicity. The 1250 W L⁻¹ treatment for 15 min, for example, caused no significant reduction in survival at the tested concentrations. The 2500 W L⁻¹ treatment for 15 min was less toxic than its 5 min counterpart, inducing significant mortality only at a concentration of 100 μg mL^−1^. These results suggest a complex, non‐linear relationship between sonication parameters and the induction of toxicity in the cashew juice matrix.

A shift in the dominant effect of ultrasound may explain the counterintuitive finding that longer sonication times reduce toxicity. The process likely involves two competing mechanisms: release and degradation. During the first few minutes of processing, the primary effect of the intense acoustic cavitation is mechanical. It breaks down plant cell structures suspended in the juice, causing the rapid release of naturally occurring, potentially toxic compounds into the liquid. This increases their effective concentration and bioavailability, resulting in the high toxicity observed in the 5 min treatments. With prolonged exposure (15 min), the process may transition from physical extraction to chemical degradation. The same sonochemical forces, including the generation of free radicals and localized high temperatures from collapsing microbubbles, may begin to degrade the compounds initially released. These molecules are likely transformed into simpler, less toxic byproducts, thereby reducing the overall toxicity of the juice.

**Figure 4:**
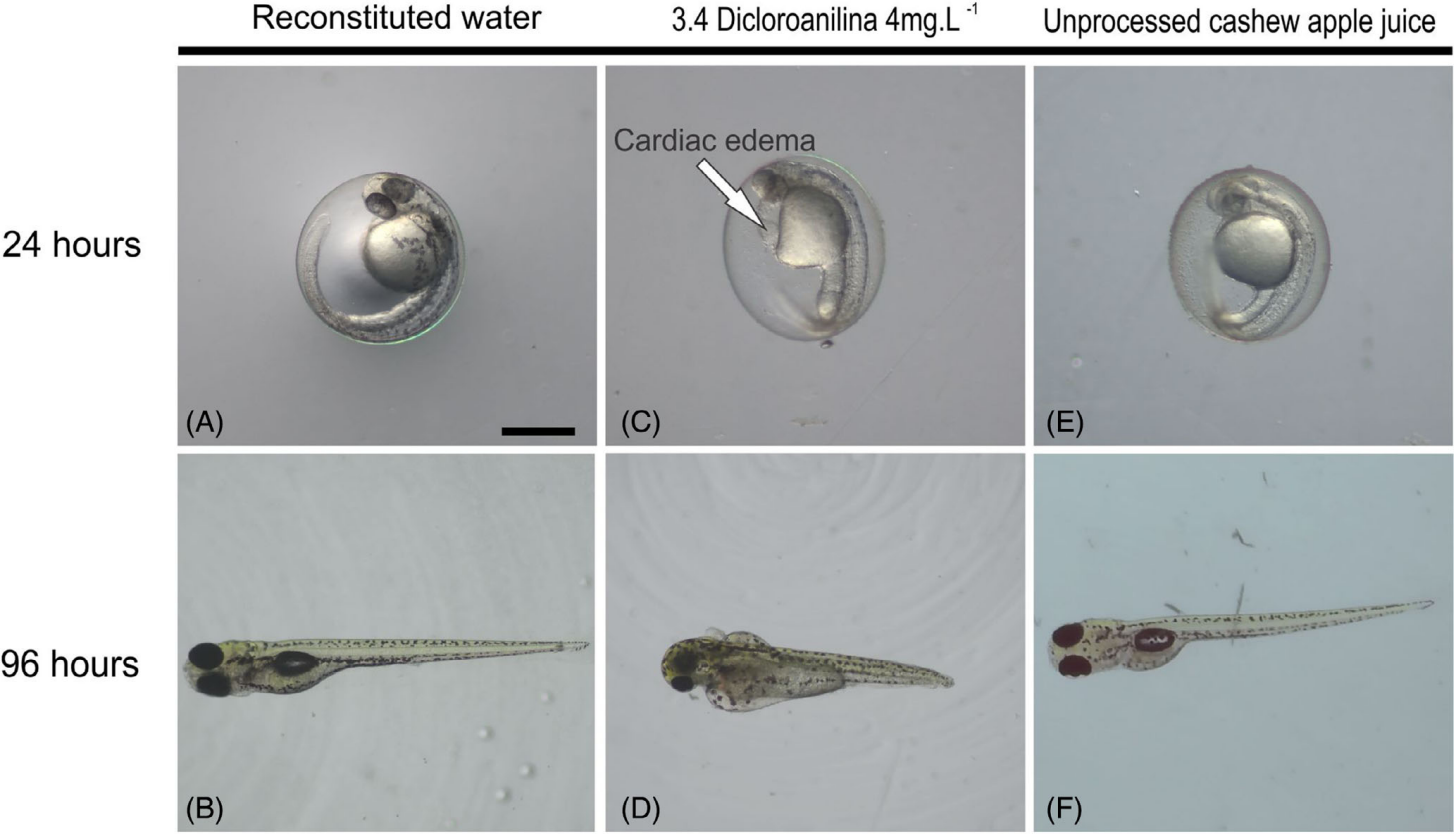
Morphology of zebrafish embryos and larvae exposed to reconstituted water for 24h (A) and 96h (B); 3,4 dicloroanilina for 24h (C) and 96h (D) and to unprocessed cashew apple juice for 24h (E) and 96h (F).

Morphologically, the embryos and larvae exposed to reconstituted water exhibited normal development, with no morphological changes (Fig. 4). The embryos exhibited intact body structures with clearly defined head, trunk, and tail regions. The body axis was straight and elongated, indicating the absence of any skeletal malformations. Typical pigmented eyes were observed. No visible swelling or fluid accumulation was detected, particularly in the pericardial region or yolk sac. The spine appeared straight, without kyphosis or lordosis, and the yolk sac was well formed, with no signs of substantial degradation or abnormality. The larvae exhibited normal, healthy development, with a straight body axis, no pericardial or yolk sac edema, and developed and intact fins. The head and eyes appeared normal, and the larvae appeared robust and appropriately sized for 96 h after fertilization.

Zebrafish embryos exposed to 3,4‐dichloroaniline showed delayed embryonic development and pronounced developmental malformations (Fig. 4). The embryos presented pronounced spinal curvature, suggesting severe skeletal development issues. A large fluid sac around the heart, larger than the head, was observed, impairing cardiac function. Severely underdeveloped or malformed head, eye, and jaw structures were also observed. The embryos appeared smaller and less developed than control embryos of the same age, indicating growth retardation. The larvae exhibited extensive malformation, confirming the assay's ability to detect toxicity. The larval body was severely curved with prominent pericardial edema. Underdeveloped fins and craniofacial defects were also observed. The larvae exhibited severely stunted growth and appeared smaller than the negative control.

The morphology of embryos and larvae exposed to unprocessed cashew apple juice also exhibited normal, healthy development, similar to that observed in embryos exposed to reconstituted water (negative control). This similarity indicates that unprocessed cashew apple juice did not induce developmental toxicity in zebrafish embryos and larvae. At the concentrations used in the experiment, the unprocessed juice was biocompatible with zebrafish development. These results suggest that the juice did not contain significant levels of compounds that interfere with fundamental biological processes necessary for normal embryonic and larval development. They also indicate that the natural constituents of cashew apple juice, at the tested concentrations, were neither acutely toxic nor teratogenic to this vertebrate model.

**Figure 5:**
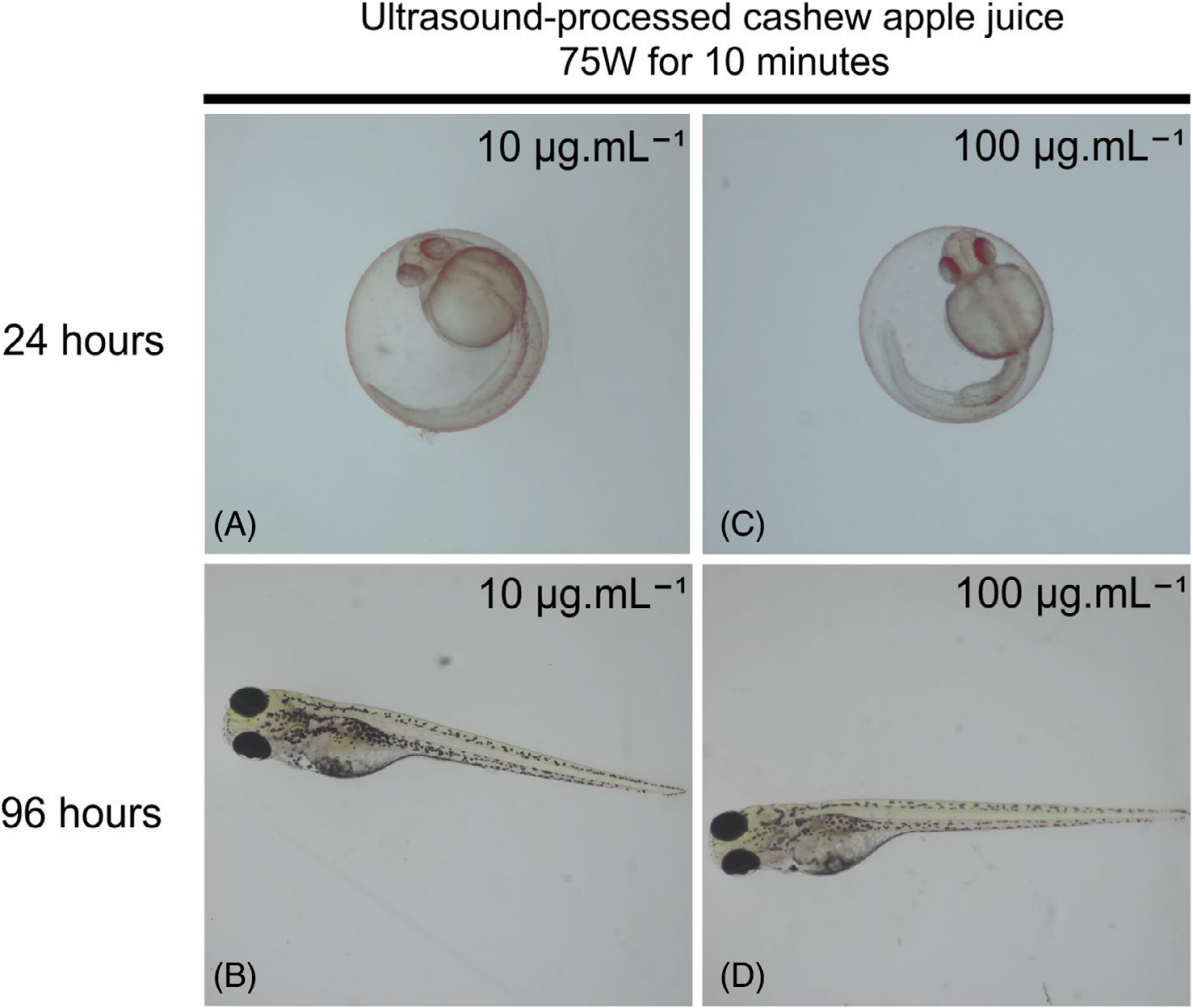
Morphology of zebrafish embryos and larvae exposed to ultrasound‐processed cashew apple juice (1875 W L⁻¹, 10 min) at 24 and 96 h of exposure.

**Figure 6:**
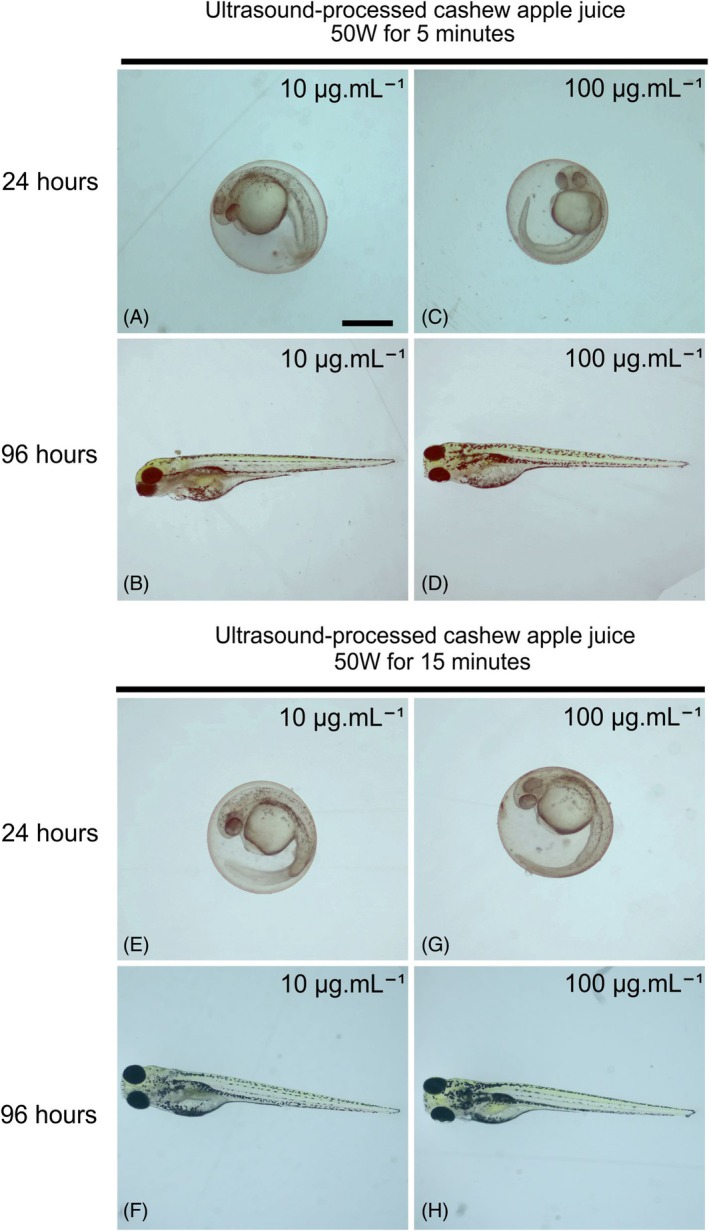
Morphology of zebrafish embryos and larvae exposed to ultrasound‐processed cashew apple juice (1250 W L⁻¹, 5 and 15 min) at 24 and 96 h of exposure.

**Figure 7:**
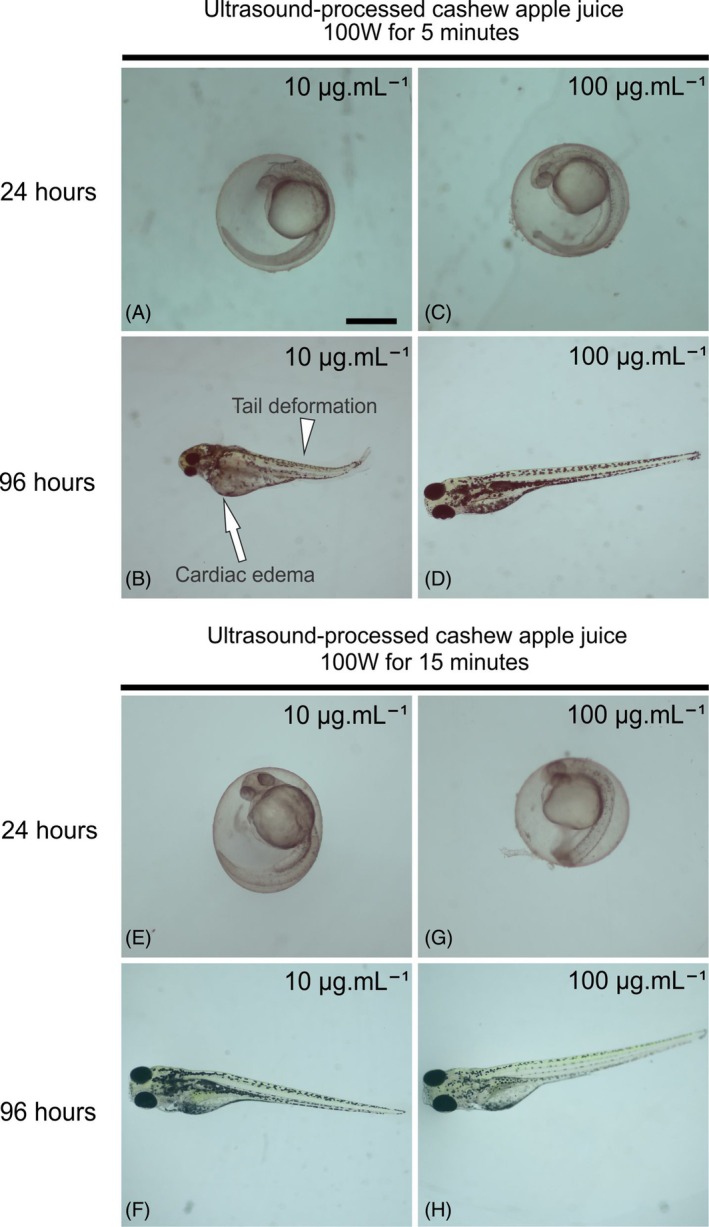
Morphology of zebrafish embryos and larvae exposed to ultrasound‐processed cashew apple juice (2500 W L⁻¹, 5 and 15 min) at 24 and 96 h of exposure.

Zebrafish embryos exposed to ultrasound‐processed cashew apple juice at concentrations of 10 and 100 μg mL^−1^ exhibited morphology similar to that of embryos exposed to reconstituted water. The chorion remained intact, and the embryos developed without morphological damage (Figs 5, 6, and 7).

Larvae exposed to sonicated juice exhibited a range of malformations, typically less severe than those observed in the positive control but clearly distinct from those in the negative control and unprocessed juice groups. The most significant and varied effects were observed in these larvae and depended on the sonication parameters.

Larvae exposed to cashew apple juice sonicated for 5 min often exhibited mild to moderate pericardial edema and occasional slight spinal curvature. Their overall size was slightly reduced compared to the controls. The effects were more pronounced at 100 μg mL⁻¹ than at 10 μg mL⁻¹. At the highest ultrasound power density (2500 W L⁻¹) more pronounced malformations were observed. Larvae exhibited significant pericardial and yolk sac edema, more noticeable spinal curvature, and stunted growth.

When larvae were exposed to cashew apple juice sonicated for longer periods (10 and 15 min), the effects were generally less severe than those observed in the 5 min treatments. Many larvae appeared close to normal, with only occasional mild edema or very subtle spinal curvature. This observation is consistent with the reduced mortality observed at longer processing times. Although not as severely malformed as those in the positive control, larvae exposed to sonicated cashew apple juice at a concentration of 100 μg mL⁻¹ exhibited more noticeable pericardial edema and some spinal curvature. They also tended to be smaller than the controls, suggesting growth retardation.

The detailed morphological analysis of zebrafish larvae in Figs 5, 6, and 7 provides visual evidence that ultrasound processing can induce developmental toxicity in cashew apple juice, with severity dependent on the specific sonication parameters employed. This conclusion is strongly supported by the developmental anomalies observed across the different treatment groups.

For this analysis, the unprocessed cashew apple juice serves as a baseline for comparison. Larvae exposed to it exhibited morphology indistinguishable from the negative control (reconstituted water). Both displayed characteristics of healthy development, including a straight body axis, fully inflated swim bladders, well formed fins, and a complete absence of edema or craniofacial defects. This confirms that the inherent chemical composition of the unprocessed juice is not toxic to zebrafish development under the experimental conditions.

In contrast, larvae exposed to ultrasound‐processed cashew apple juice consistently exhibited a range of dose‐ and parameter‐dependent malformations. These alterations exceeded the normal variation observed in controls and fall within the recognized spectrum of developmental toxicity in zebrafish.

Larvae exposed to many sonicated conditions, particularly those related to short sonication times, showed visible pericardial edema. In the most severe cases, this manifested as pronounced swelling around the heart, which directly impeded proper circulation and nutrient delivery, leading to broader developmental issues. Yolk sac edema was also observed, indicating disrupted yolk absorption and overall physiological stress.

Compared with the severe C‐shapes induced by the positive control, many sonicated samples displayed mild to moderate spinal curvatures. This subtle yet distinct bending of the body axis suggested disruptions in notochord formation or muscle development, which are critical processes sensitive to chemical stressors.

Larvae from several sonicated groups, especially those exposed to high power density (2500 W L⁻¹) and short processing times, appeared visibly smaller and less robust than their unprocessed counterparts at 96 hpf. This observation indicates that the developmental process was inhibited, likely due to metabolic disruption or cellular stress caused by compounds present in the processed juice. Less severe than the spinal or edema‐related effects, subtle abnormalities in head shape and eye size were observed in the sonicated groups, suggesting disruptions in fundamental anterior–posterior patterning.

The severity of these morphological abnormalities varied significantly with the sonication parameters, reinforcing the idea that the processing itself was a source of toxicity. For example, the 2500 W L⁻¹, 5 min treatment induced more pronounced edema and spinal defects than the 1250 W L⁻¹, 5 min treatment, indicating a power‐dependent effect. The observation that longer duration treatments resulted in larvae appearing near normal, with minimal or no observable malformations, supports the hypothesis of a complex interplay between the release and subsequent degradation of toxic compounds during sonication.

The total length of zebrafish larvae and yolk sac size after 96 h. The exposure to the negative control (reconstituted water) and the unprocessed cashew apple juice resulted in the normal, expected larval length above 3.5 mm. In contrast, exposure to the positive control resulted in stunted growth, with larvae reaching only 3.3 mm in length. Larvae exposed to the various sonicated cashew apple juices showed no significant negative impact on length. Across all tested concentrations (10 and 100 μg mL^−1^), larvae grew to lengths comparable with the negative control group.

The size of the yolk sac remaining after 96 h is an indicator of nutrient absorption efficiency during larval growth. A larger remaining yolk sac suggests delayed or impaired development. As expected, the positive control resulted in the largest average yolk sac size and the negative control resulted in a smaller yolk sac. Exposure to unprocessed cashew apple juice was not significantly different from the negative control, indicating normal nutrient absorption (data not shown).

### Considerations regarding the toxicity tests

This study revealed a considerable contrast in results between the two biological models, with zebrafish proving to be a significantly more sensitive indicator of toxicity for ultrasound‐processed cashew apple juice than the brine shrimp, *A. salina*. The fundamental difference was that cashew apple juice, whether unprocessed or sonicated, showed no significant toxicity towards *Artemia salina* nauplii. In contrast, the ultrasound‐processed juice induced significant, dose‐dependent toxic effects in zebrafish embryos and larvae, including mortality and mild developmental malformations.

Regarding the unprocessed cashew apple juice, tests with *A. salina* did not reveal toxicity, with mortality rates comparable to those of the control group. The toxicological tests with zebrafish also found the unprocessed juice to be biocompatible, resulting in nearly 100% survival at 24 h and normal, healthy development similar to the negative control. These results indicate that the unprocessed cashew apple juice is not the primary source of toxicity.

Regarding the sonicated cashew apple juice, *A. salina* remained unaffected. Across all tested power densities and durations, mortality rates were low and did not differ significantly from those of the control group. This suggests the processing did not create compounds that were lethal to this organism. However, the sonicated juice showed significant toxicity toward zebrafish, which was dependent on sonication time and power. The observed effects were both lethal and sublethal, the latter including developmental abnormalities, cardiac and yolk sac edema, skeletal malformations, and growth retardation. This suggests that processing created compounds toxic to this organism.

*Artemia salina* is a robust and relatively simple organism, and the toxicological tests using this species primarily measure acute lethality. This model may lack the complex physiological and molecular pathways needed to detect the sublethal toxic effects observed in zebrafish. Its ability to adapt to unfavorable environments through osmoregulation may also contribute to its resistance. Zebrafish, as a vertebrate model, are far more sensitive. They share approximately 70% of their protein‐coding genes with humans, making them an excellent model for developmental toxicology. The transparent embryos enable detailed morphological analysis, revealing sublethal and teratogenic effects that a simple mortality count in *A. salina* would miss.^22^, ^23^

## DISCUSSION

The toxicological implications of high‐intensity ultrasound on food matrices are not fully understood, despite its widespread application in food preservation. In the present study, *A. salina* assays failed to detect toxicity in sonicated cashew apple juice, whereas *D. rerio* models revealed significant developmental anomalies dependent on sonication power and duration. This discrepancy highlights the limitations of simple invertebrate models for food safety assessment and supports the hypothesis that sonication parameters critically modulate the release and degradation of toxic compounds.

The observed lack of toxicity in *A. salina* suggests that the ultrasound parameters used may not have been sufficient to generate harmful byproducts in the juice at concentrations toxic to this model organism. Ultrasound can also degrade undesirable compounds, such as anacardic acids,^9^ which may have contributed to this result.^24^

The lack of toxicity from unprocessed cashew apple juice at low concentrations is consistent with previous findings.^5^ The absence of toxicity in sonicated cashew apple juice is also consistent with research on other plant‐based matrices subjected to non‐thermal processing. For example, a study on various avocado extracts^25^ also reported a lack of toxicity. Similarly, coconut water processed by ultrasound and cold plasma showed no adverse effects on *A. salina*.^25^ However, the safety of non‐thermally processed foods is highly dependent on both the food matrix and the specific technology used. This is illustrated by a contrasting study in which coconut water treated with ozone, another non‐thermal method, induced toxicity in *A. salina*.^25^ This variation highlights a critical point: although ultrasound did not produce toxic effects in cashew juice detectable by *A. salina*, other processes or food substrates may yield different results.^26^, ^27^, ^28^

Although no acute toxicity was observed in the present study, ultrasound processing is known to alter the chemical composition of cashew juice, for example by increasing the concentration of anacardic acid, as reported previously.^9^ The absence of mortality in the robust *A. salina* model therefore highlights the importance of using multiple biological models to conduct a thorough safety assessment.

The toxicity observed during the initial minutes of processing may be linked to the release of anacardic acids. These phenolic lipids are toxic in animal studies,^29^, ^30^ and their concentration increases in ultrasonically treated cashew juice, as previously reported by Alves Filho *et al*.,^9^ directly linking sonication to a higher concentration of a known toxin. Based on the available evidence, anacardic acid is the compound most likely to undergo degradation during prolonged ultrasound exposure, as it is susceptible to chemical scission. The sonication process may therefore initially enhance the extraction of anacardic acid from the juice matrix, accounting for the toxicity observed after short processing times.^31^ As treatment continues, the sustained high energy of the sonochemical environment may degrade the complex structure of the anacardic acid molecule itself, reducing its concentration and thereby decreasing the juice's overall toxicity.^32^

The pronounced morphological damage observed in zebrafish larvae exposed to ultrasound‐processed cashew apple juice, including pericardial edema, spinal curvature, and growth retardation, strongly suggests underlying disruptions at the cellular and genetic levels. The edema, particularly in the pericardial region, suggests potential impairment of the cardiovascular system. This observation aligns with previous research indicating that toxicological insults can impact heart development, potentially through alterations in genes such as heart and neural crest derivatives expressed 2 (*hand2*), hyaluronan synthase 2 (*has2*), and southpaw (*spaw*), which are fundamental for cardiac differentiation and function.^33^, ^34^

The spinal curvature and overall growth retardation observed in the present study are consistent with disruptions in notochord development and cell cycle regulation. Jerafi‐Vider *et al*.^33^ linked such developmental damage in zebrafish to changes in gene expression governing cell cycle progression and notochord integrity. This mechanism was further supported by Kim *et al*.,^35^ who demonstrated that the herbicide benfluralin induced transcriptional changes in genes related to cell cycle and notochord development, resulting in growth retardation and toxicity, as observed in the present zebrafish model.

The spectrum of damage reported for ultrasound‐processed cashew apple juice, including growth retardation, malformations, and edema, is also a common signature of developmental toxicity described in numerous studies using zebrafish. Several reports in the literature document similar morphological changes resulting from exposure to diverse contaminants, including nanoparticles,^36^ pesticides,^12^, ^37^ and microplastics.^38^ Several food‐related substances, including additives and preservatives, have been shown to induce comparable effects in zebrafish. For example, Leonard *et al*.^39^ found that *tert*‐butylhydroquinone (tBHQ) negatively impacted survival and growth, while nitrite,^40^ Sunset Yellow (E110), and tartrazine^41^, ^42^ all caused yolk sac edema and other malformations. The inhibition of swim bladder development by erythrosine dye^43^ further shows the sensitivity of zebrafish to diet‐related stressors.

## CONCLUSION

This study demonstrated that, although unprocessed cashew apple juice is biocompatible, the application of high‐intensity ultrasound can induce significant developmental toxicity in a vertebrate model. The results reveal a complex, non‐linear relationship in which short sonication times were more toxic than prolonged exposure. This observation is probably caused by a dual‐effect mechanism involving an initial release of naturally occurring toxic compounds, such as anacardic acids, followed by their subsequent chemical degradation with continued sonication.

Based on the toxicological tests, longer sonication times (15 min) were significantly safer than short (5 min) treatments, resulting in less severe effects and higher survival rates. Specifically, the treatment at 1250 W L⁻¹ for 15 min caused no significant reduction in larval survival and was the safest processing condition identified in this study. This suggests that allowing the sonochemical process sufficient time to degrade the initially released toxins is a more effective strategy for ensuring safety.

These results have important implications for the food industry, challenging the assumption that non‐thermal processing is inherently safer than conventional thermal methods. The optimization of technologies such as ultrasonication must therefore balance the preservation of nutritional and sensory qualities against the potential release of bioactive compounds that may pose a food safety risk.

The contrasting results obtained from the *A. salina* and zebrafish toxicological tests highlight the limitations of simple invertebrate mortality assays, such as those using *A. salina*, for evaluating novel food products. The severe, sublethal teratogenic effects observed in zebrafish demonstrate the need for sensitive, genetically relevant vertebrate models in regulatory safety assessments.

## Data Availability

The data that support the findings of this study are available on request from the corresponding author. The data are not publicly available due to privacy or ethical restrictions.
